# Surgical Management of Pediatric Capitellar Fractures: A Case Series of Five Patients

**DOI:** 10.7759/cureus.88605

**Published:** 2025-07-23

**Authors:** Eleni Pappa, Anastasia Pilichou, Konstantinos Neroutsos, Maria Giannakopoulou, Spyridon Maris, Panagiotis Tsintavis, John Anastasopoulos

**Affiliations:** 1 5th Orthopaedic Department, KAT General Hospital, Athens, GRC; 2 Orthopaedics and Trauma, 2nd Department of Paediatric Orthopaedics, Agia Sofia Children's Hospital, Athens, GRC

**Keywords:** capitellum fractures, elbow fractures, elbow trauma, open reduction internal fixation, pediatric orthopedics

## Abstract

Capitellum fractures are rare in pediatric patients and pose unique diagnostic and management challenges due to the presence of developing ossification centers and growth plates. Timely recognition and appropriate treatment are essential to prevent long-term complications such as stiffness, avascular necrosis, or growth disturbances. This case series presents five pediatric patients with capitellum fractures, classified as Dubberley type 2A, detailing their surgical management and their postoperative outcomes. The cases were treated at a single institution over a two-year period. Fractures were classified based on imaging, including plain radiographs and, where needed, computed tomography (CT). The treatment strategy included open reduction and internal fixation with headless screws through a posterolateral approach. The five cases involved patients aged between 11 and 14 years, with mechanisms of injury including falls from height and sports-related trauma. All patients achieved satisfactory functional outcomes, with no reported cases of avascular necrosis or significant growth disturbances at a mean follow-up of 8.8 months. Minor complications included transient stiffness in two patients, which resolved with physiotherapy. In conclusion, capitellum fractures in children, though uncommon, require high clinical suspicion and appropriate imaging for accurate diagnosis. Early and individualized management, tailored to the fracture type and patient needs, can yield excellent outcomes. This case series underscores the importance of a multidisciplinary approach in optimizing care for pediatric elbow injuries.

## Introduction

Capitellar fractures are uncommon in the pediatric population, representing only a small percentage of overall elbow injuries [1]. Specifically, coronal shear fractures of the distal humerus make up approximately 1% of all elbow fractures in the general population and account for 3% to 6% of fractures involving the distal humerus [2]. Although these injuries are rare, they are clinically important due to their potential impact on joint mechanics and skeletal development.

In children, the development of the capitellum is influenced by complex growth patterns involving multiple ossification centers and growth plates. The elbow contains six such centers, which ossify and fuse in a consistent sequence commonly remembered by the acronym CRITOE, referring to the capitellum, radial head, internal (medial) epicondyle, trochlea, olecranon, and external (lateral) epicondyle [3, 4]. Before the complete fusion of these centers during adolescence, any trauma to the region can result in chondral or osteochondral injuries that may go undetected on standard radiographs, particularly if the fragments are small [5]. These fractures are most often seen in adolescents nearing the age of 14, which coincides with the late stages of ossification [6].

The mechanisms responsible for these injuries in pediatric patients differ somewhat from those seen in adults. While adult capitellar fractures are typically caused by direct trauma or shear forces, children are more frequently injured during high-energy activities such as sports or falls [7]. Two distinct injury mechanisms have been identified. The first involves indirect axial loading through the extended arm, which generates vertical shear stress on the distal humerus via the radial head [8]. The second mechanism occurs during the reduction of an elbow that has been dislocated or subluxated posterolaterally. In such cases, the radial head and coronoid process can impact the capitellum, leading to a shear fracture [9].

Clinically, affected patients may present with localized pain, swelling, and limited elbow mobility. However, the subtle nature of these injuries on imaging often delays diagnosis. A high degree of clinical suspicion is required, especially since standard radiographs may reveal only indirect signs such as a fat pad sign in the presence of small chondral fragments or a double contour sign when the subchondral bone is involved [10]. These fractures are often considered part of a group of injuries that appear relatively benign on imaging, necessitating advanced diagnostic tools such as CT scans to confirm the extent of the lesion [11].

Various classification systems have been developed to better characterize capitellar shear fractures in both children and adults. The modified Bryan-Morrey classification outlines three main fracture types: the first type involves the entire capitellum; the second refers to an osteochondral injury of the anterior articular surface with minimal subchondral bone; and the third describes a comminuted or compressive fracture. A fourth type, introduced by McKee, incorporates fractures that extend into the trochlea [12, 13, 14]. In contrast, the Dubberley classification, arguably the most widely used, categorizes fractures into three main types based on the degree of articular involvement. The first type includes fractures confined to the capitellum, sometimes involving the lateral trochlear ridge; the second type involves the capitellum and trochlea as a single unit; and the third includes fractures where the capitellum and trochlea are separated into distinct fragments. Each type is further classified according to the presence or absence of posterior condylar comminution. This system has greater prognostic relevance, as it has been shown that fractures with minimal fragmentation and no posterior involvement are associated with improved functional outcomes [15].

Treatment of capitellar fractures in children presents unique challenges due to the growing nature of the skeletal system. The primary goals of management include preserving the growth plate, maintaining joint congruency, and minimizing the risk of long-term complications such as avascular necrosis, joint stiffness, or deformities resulting from growth disturbances. Non-operative management, such as immobilization in a long-arm cast, has historically been used for nondisplaced fractures. However, recent evidence suggests that conservative approaches often yield unsatisfactory results and are now rarely used [6].

In specific scenarios where the fracture is isolated, comminuted, non-reconstructable, does not involve the trochlea, and the medial collateral ligament remains intact, fragment excision may be considered [16]. Nonetheless, for the majority of displaced fractures, the current standard of care is open reduction and internal fixation, which allows for accurate anatomical restoration and early mobilization, reducing the likelihood of complications [2].

This case series presents our departmental experience with the surgical treatment of capitellar fractures in pediatric patients. By evaluating the functional outcomes of these cases, we aim to contribute valuable clinical insights and help refine treatment protocols for this rare but significant injury.

## Case presentation

**Figure 1:**
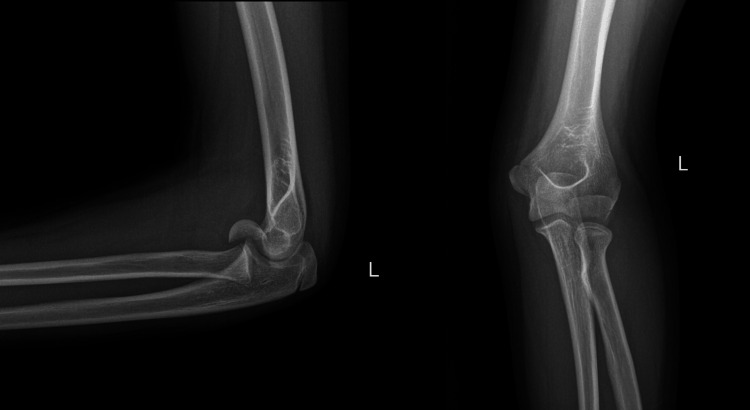
Preoperative anteroposterior and lateral radiograph depicting the capitellar fracture. The double arc sign is present. The olecranon and the medial epicondyle have not been fused yet.

**Figure 2:**
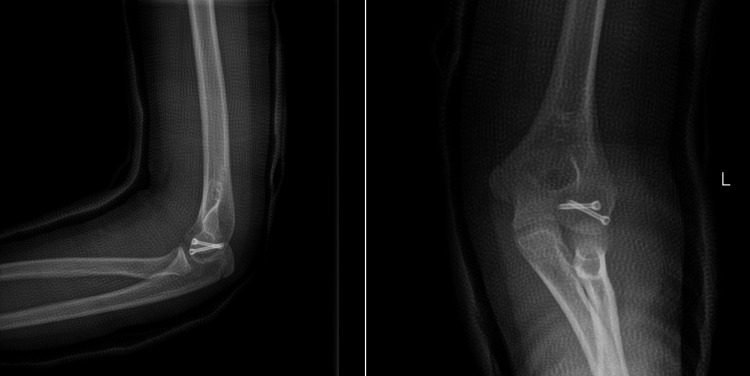
Anteroposterior and lateral radiographs of the open reduction and internal fixation of the capitellar fracture with headless screws were obtained two weeks postoperatively.

**Figure 3:**
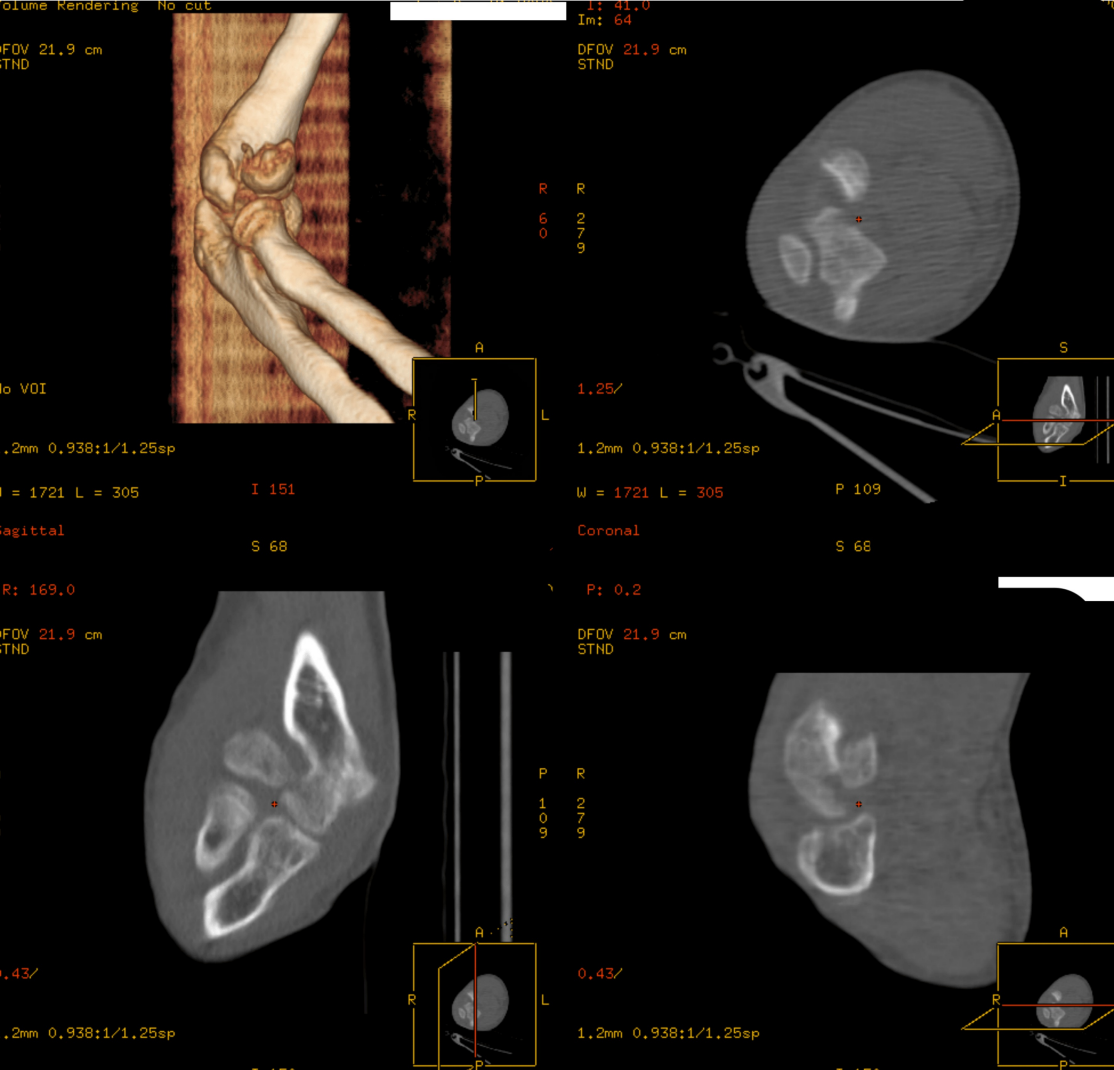
Preoperative CT assessment of the capitellar fracture.

This retrospective case series was initiated following institutional review board approval. From December 2022 to January 2024, five pediatric patients were presented to our department with a Dubberlay type 2A capitellum fracture, who underwent surgical treatment with headless compression screws. Preoperative and postoperative radiographic images, as well as additional imaging such as CT, were obtained, along with informed consent. All of the patients had adequate clinical and radiographic follow-up and were evaluated for the results of the treatment. Medical records of all the patients were reviewed in order to determine demographic data, mechanism of injury, surgical technique, and elbow postoperative range of motion, as well as functional postoperative results according to the Disabilities of the Arm, Shoulder, and Hand (DASH) questionnaire [17]. Complications such as lack of motion, symptomatic implants, or any postoperative complications, such as neurovascular injury or infection, were also noted on follow-up, as well as the radiographic presence of avascular necrosis. In our case series, the mean patient age at the time of injury was 13.2 years (range 11 to 14 years old), with three of them being males and two females (Table 1, Figures 1-3).

**Table 1:** The Dubberley classification of capitellar and trochlear fractures Source: [13]

Type / Subtype	Description
Type 1	Fracture involves primarily the capitellum, may extend minimally into the trochlea.
Type 2	Fracture involves both the capitellum and a larger portion of the trochlea as a single piece.
Type 3	Fracture involves separate fragments of the capitellum and trochlea (they are not a single piece).
Subtype A	Fracture without posterior condylar comminution.
Subtype B	Fracture with posterior condylar comminution.

The operation was carried out by a single pediatric orthopedic surgeon. A lateral approach to the elbow was performed. The Kaplan interval between the extensor carpi radialis brevis and the extensor digitorum communis was used. The common extensor origin was then released, and an arthrotomy of the radiocapitellar joint was performed, always with the forearm in pronation in order to protect the posterior interosseous nerve. The capitellar fracture was reduced under direct vision, and the optimal reduction was confirmed by intraoperative fluoroscopy through a mini C-ARM. Internal fixation was achieved with two headless compression Herbert screws in all of the patients in an anterior-to-posterior configuration. There were no coexisting bony or ligamentous injuries. Regarding the postoperative rehabilitation protocol, the elbow was put in a cast for four weeks, with the immediate start of range of motion and strengthening physiotherapy afterwards. Follow-up was a minimum of 12 months in all five cases.

Results

All patients presented with a Dubberley type 2A fracture of the capitellum and were operatively treated within one week of their initial trauma. All fractures were healed in a mean of five weeks (range, four to six weeks), as confirmed by plain X-rays.

**Figure 4:**
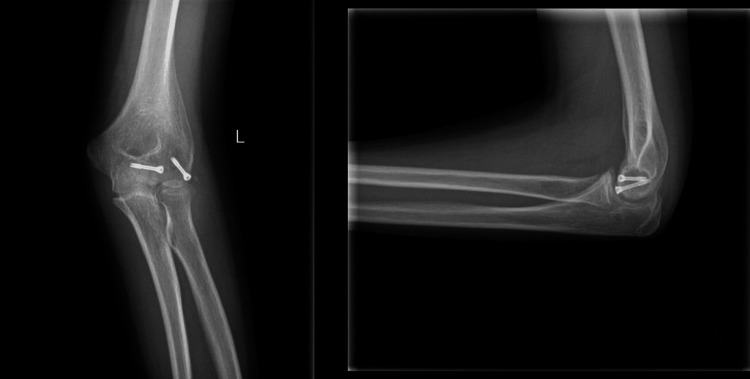
Postoperative anteroposterior and lateral radiograph on the last follow-up, six months postoperatively.

At the end of the follow-up, all the patients achieved fracture healing without any malunion or pseudarthrosis present, nor any osteonecrosis. Also, it is worth mentioning that all the patients after the stitches removal, which took place 15 days postoperatively, were already presenting with a painless range of motion, which was also present during the last follow-up postoperatively. In comparison with the range of motion of the contralateral elbow, there was a full range of motion achieved with an arc of 140 degrees of flexion and five degrees of extension. However, in only one patient was there a lack of extension of 12 degrees, whereas in another, there was a flexion, a lack of motion of 15 degrees. Pronosupination was intact. There were no neurovascular injuries or infections present during the last follow-up. The mean DASH score was 12, with a range of five to 60. All the patients returned to their pre-injury level of daily activity (Figure 4).

## Discussion

Capitellum fractures are infrequent and present notable challenges in both diagnosis and treatment. The existing literature is predominantly composed of isolated case reports and a limited number of retrospective case series [18], which contributes to ongoing debate regarding the optimal management approach. Despite the limited evidence base, open reduction and internal fixation remains the most commonly recommended treatment for coronal shear fractures of the capitellum [19].

A range of fixation methods have been described in the literature, including Kirschner wires, AO screws, bioabsorbable pins, headless compression screws, and osteosutures [13, 16, 20, 21]. Among these, Herbert's headless compression screws have emerged as the preferred implant, especially for fractures limited to the anterior articular surface [22]. These screws are designed to sit flush beneath the cartilage, minimizing the risk of joint surface irritation, and they provide strong compression at the fracture site. In our series, Herbert screws of variable sizes were used in all cases, inserted from anterior to posterior in a perpendicular orientation relative to the fracture plane.

Alternative fixation strategies, such as buttress plating, low-profile locking plates, or even hinged external fixators, are typically reserved for more complex injury patterns involving disruption of the posterior capitellar cortex or concomitant ligamentous and osseous injuries, such as collateral ligament tears or associated fractures of the radial head or humeral epicondyles [21].

Exposure of the fracture site can be technically demanding, particularly in cases with significant medial extension. The choice of surgical approach is largely dictated by the fracture morphology. The lateral and anterolateral exposures are the most frequently utilized techniques. When the capitellar fracture extends into the trochlea in a continuous fragment, either an extensile lateral or an anterolateral approach may be appropriate. However, in cases where the capitellum and trochlea are fractured as separate fragments and the posterior column remains intact, the anterolateral or a bilateral approach is often more suitable [23, 24].

In selected cases with broad articular involvement or medial epicondyle fractures, a posterior transolecranon approach may be indicated. Nonetheless, this route poses a risk to the posterior vascular supply of the capitellum, which may compromise healing. In our cohort, we employed the lateral approach using the Kaplan interval for all surgical exposures.

Postoperative recovery is typically uncomplicated in most patients. However, several factors have been shown to negatively influence clinical outcomes. Chief among these are extensive articular fragmentation and posterior comminution. Studies have demonstrated that Dubberley type 2 and type 3 fractures are associated with poorer outcomes, including reduced range of motion and lower elbow function scores, compared to type 1 fractures [13, 16, 25]. Additionally, fractures without posterior comminution (classified as Dubberley type A) tend to have better functional recovery than those with comminution (type B) [26, 27].

Complications following surgery are relatively infrequent. The most commonly reported indications for revision surgery include elbow contracture and symptomatic implants. Other potential complications include nonunion, osteonecrosis, heterotopic ossification, post-traumatic arthritis, ulnar nerve symptoms, radial nerve injuries, and infections. Among these, posttraumatic arthritis is particularly impactful, as it can significantly diminish long-term elbow function and patient-reported outcomes [13, 14, 22]. Achieving accurate anatomic reduction is essential in minimizing the risk of post-traumatic arthritis. Although some concerns exist regarding chondral damage from anterior-to-posterior screw insertion, comparative studies have found no significant difference in outcomes between anterior-to-posterior and posterior-to-anterior screw trajectories [28].

In our series, no major postoperative complications were observed throughout the follow-up period. Minor limitations in elbow flexion and extension were noted in two patients, respectively.

Nonetheless, our study is not without limitations. As a retrospective case series, it inherently carries the limitations of observational research. Additionally, our follow-up duration was shorter compared to other published studies. Furthermore, the sample size of our cohort was relatively small. Despite this, to our knowledge, this represents the largest reported case series of pediatric capitellar fractures in the Greek population; however, due to the lack of a current registry of these specific injuries, more research on the management and functional results of this injury needs to be conducted.

## Conclusions

Pediatric capitellar fractures are rare injuries that are likely to have poor outcomes if left undiagnosed and untreated. Early diagnosis and anatomic reduction are essential for a successful outcome. Treatment with open reduction and internal fixation with headless Herbert screws provides a stable fixation and obtains satisfactory postoperative functional results. However, as our case series includes only five patients, we believe that future research should prospectively study these pediatric fractures, not only to draw decisional trees but also to find out the predictors of outcome, especially for the development of severe sequelae such as radiocapitellar osteoarthritis and osteonecrosis of the capitellum, which can all be diminished with timely and correct diagnosis and treatment.
